# Evolution of EBV seroprevalence and primary infection age in a French hospital and a city laboratory network, 2000–2016

**DOI:** 10.1371/journal.pone.0175574

**Published:** 2017-04-17

**Authors:** Grégoire Fourcade, Raphaele Germi, Fabrice Guerber, Julien Lupo, Monique Baccard, Arnaud Seigneurin, Touyana Semenova, Patrice Morand, Olivier Epaulard

**Affiliations:** 1 Infectious Disease Unit, Grenoble University Hospital, Grenoble, France; 2 Fédération d’Infectiologie Multidisciplinaire de l’Arc Alpin, Université Grenoble Alpes, France; 3 Laboratory of Virology, Grenoble University Hospital, Grenoble, France; 4 Team “HIV and other persistent human viruses”, Institut de Biologie Structurale, UMR5075 CEA-CNRS-UGA, Grenoble, France; 5 Oriade Laboratory, Vizille, France; 6 Unité d'évaluation médicale, Grenoble University Hospital, Grenoble, France; 7 Techniques de l'Ingénierie Médicale et de la Complexité - Informatique Mathématiques et Applications Grenoble, Unité Mixte de Recherche, Grenoble, France; University of North Carolina at Chapel Hill, UNITED STATES

## Abstract

**Background:**

According to rare studies, the age at EBV primary infection (PI) has recently risen in some developed countries. A later age at infection is generally considered a risk factor for severe EBV PI, although few studies exist on this subject. Our investigation aimed to determine whether EBV seroprevalence and EBV PI epidemiology have evolved in France, and to what extent age and infection intensity (regarding biological parameters) are correlated.

**Methods and findings:**

We conducted a retrospective study of the following EBV serological tests databases: tests carried out at Grenoble University Hospital (2000–2016) (n = 53,553); and tests carried out by a network of city laboratories in Grenoble area (2008–2015) (n = 27,485). The hospital population showed a continuous, significant decrease in EBV seroprevalence over the studied period for patients aged 20 and over (p<0.01). The seroprevalence also decreased for different age classes (<10, 15–19, 20–30, and 30–40 years old) over the periods 2001–2005, 2006–2010, and 2011–2015. Consistently, the age at PI was significantly higher in the years 2008–2015 than in the years 2001–2007 (15.6±12.0 vs. 13.7±11.0; p = 0.03). The city laboratory population showed the same trend of decreasing seroprevalence (p = 0.06); no significant variations in age at PI were observed. The age at PI was positively correlated with ASAT, ALAT, γGT, and bilirubin blood levels (p<0.01) and negatively correlated with platelet counts (p<0.05).

**Conclusion:**

In the last 15 years, the age at EBV PI has increased, whereas seroprevalence has decreased. Moreover, our findings confirm the positive correlation between age and biological abnormalities. Taken together, these results suggest that the incidence of severe EBV PI will increase in the future.

## Introduction

Infectious mononucleosis (IM) is the symptomatic form of Epstein–Barr Virus (EBV) primary infection (PI). IM primarily occurs in adolescents or young adults: up to 77% of EBV PIs are clinically symptomatic in patients aged 18–22 [1], whereas EBV PI in childhood is generally associated with few or no symptoms. IM may require hospitalization due to either benign (acute dysphagia, acute fatigue, hepatitis, etc.) or severe (hemophagocytic syndrome, encephalitis, etc.) disorders. IM is traditionally considered to intensify with age of onset, although only few studies have explored this link [2].

More than 90% of the world population is infected with EBV [3]; some studies have suggested that the age at EBV PI has risen in recent years. Indeed, a study of 6-to-19-year-olds conducted in the USA shown that seroprevalence declined from 72% in the years 2003–2004 to 65% in the years 2009–2010 [4]. A study conducted in Japan [5] has shown that before the early 1990s, more than 80% of children aged 5 to 7 were found to be seropositive, whereas the positivity rate decreased to 59% for the years 1995–1999. In contrast, in Finland, EBV seroprevalence in pregnant women has remained unchanged in the last 20 years [6]. This age shift observed in some countries could lead to a higher incidence of symptomatic EBV PI, particularly of intense IM.

To determine whether the age at EBV PI in our geographical region (*Département de l’Isère*, France) has changed over the last 15 years, it would be necessary to perform an EBV serology in all the population, or to at least a random sample of this population; however, such a screening is not performed. We therefore chose to conduct a retrospective study of EBV infection (seroprevalence, and age at EBV PI) using 2 databases: 1) EBV serological tests performed at Grenoble University Hospital and 2) EBV serological tests performed by various private laboratories in the same region. We also investigated the relationship between patient age and PI-associated biological disorders in the hospital database. Although the choice of such databases may expose to biases (EBV tests were performed for diagnosis purpose, not to explore seroprevalence), we considered that they were an acceptable compromise.

## Materials and methods

### Population and data

No systematic EBV infection screening is performed in the general population. We therefore analyzed 2 EBV serological test databases:

1. a database composed of the results of all the 53,553 serological tests performed in Grenoble University Hospital between 2000 and the first quarter of 2016 in hospitalized and non-hospitalized patients;
2. A database composed of the results of all the 27,485 serological tests performed in a network of up to 27 private city laboratories (Oriade-Noviale, gathering 20% of the Isère laboratories) between 2008 and 2015.

We chose to use EBV serological results that were available regardless of the initial reason for testing; given this absence of filtering criteria, we assumed that the patients might be at least partially representative of the general population. The following variables were available for each serological record: IgM anti-VCA, IgG anti-VCA, and IgG anti-EBNA levels, and (inconstantly) heterophile antibody test results, and the patient’s date of birth, the date of serological testing, and (for the hospital database) the patient’s name.

For the hospital population, sera were tested for VCA IgM and VCA IgG with the Enzygnost anti-EBV/IgM II and the Enzygnost anti-EBV/IgG reagents (Siemens Healthcare Diagnostics, Marburg, Germany), for EBNA antibodies using the BMD EBV EBNA-IgG reagent (BioMedical Diagnostics, Marne la Vallée, France), and for heterophile antibodies with the MonoSpot assay (Meridian Life Science, Memphis, Tennessee, USA). For city laboratory population, sera were tested for VCA IgM, VCA IgG and EBNA IgG with the Liaison assay (DiaSorin, Torino, Italia).

We excluded cases for which it was impossible to determine the patient age or EBV serological status, as well as (for primary infection study) serological tests obtained for patients hospitalized in other institutions (n = 3,527 serological results), as the accuracy of the data of these patients may be questionable.

We first determined the proportion of seropositive patients for each year (seroprevalence study). We then analyzed the population of patients with EBV PI to determine whether the age at EBV PI varied over the studied period. Finally, we collected several biological data (see lower) from the medical records of patients with an EBV PI available in the hospital database to determine whether age and PI intensity were correlated.

### Seroprevalence study

The proportion of seronegative patients (in patients aged 20 and over; and in 6 different age classes) was determined for each year between 2001 and 2015 (hospital database) and between 2008 and 2015 (city laboratory database). The years 2000 and 2016 of the hospital database were excluded from the seroprevalence study because the number of serological records was low compared with the other years. We classified the patients as mentioned in Table 1.

**Table 1:** Classification of EBV status.

anti-VCA IgM	anti-VCA IgG	anti-EBNA IgG	Status
-	-	-	Seronegative
+	-	-	Primary infection
+	+	-	
-	+	-	Seropositive (past infection)
-	-	+	
-	+	+	
+	+	+	

All cases with PI (positive IgM anti-VCA without positive IgG anti-EBNA and/or positive heterophile antibody test) were excluded from the seroprevalence study. For patients with multiple serological test results obtained over several years, all serological records were included in the analyses, with a maximum of one per year. Moreover, patients with 2 serological tests obtained in the same year that revealed different results (negative then positive) were considered to have primary infections and were excluded from the corresponding year (S1 and S2 Figs).

Noteworthy, the number of EBV serological tests performed increased over the year (2672±297 per year before 2008 and 3021±91 per year after 2007, p = 0.02).

### Primary infection study (hospital database)

Patients were considered to have a primary infection if they had positive heterophile antibody test results and/or IgM anti-VCA, without IgG anti-EBNA. All duplicates were excluded. We were able to access individual medical records, which allowed us to exclude patients with health conditions that could interfere with EBV serological testing (e.g., human immunodeficiency virus (HIV) primary infection). As EBV PI after the age of 50 is considered a rare event, we analyzed, in patients aged 50 or over with only anti-VCA IgM, the results of posterior EBV serology (when available) to determine whether it was a primary infection. If no IgG VCA or IgG EBNA appeared in the following weeks or months, we considered that the IgM result was a false positive, and excluded the subject from the primary infection study (S1 Fig).

We collected the length of hospital stay (where applicable) and the following biological data: blood aspartate aminotransferase (ASAT) (UI/L), alanine aminotransferase (ALAT) (UI/L), gamma-glutamyltransferase (γGT) (UI/L), bilirubin (μmol/L), C-reactive protein (CRP) (mg/L), neutrophils (G/L), lymphocytes (G/L and %), activated (or “atypical”) lymphocytes (%), and platelets (G/L). If several values were available, we used the “worst” value (the lower value for neutrophils and platelets, the higher value for others) if taken 1 week before or after EBV testing. We did not collect these values for patients who exhibited medical conditions that might interfere with one of the collected parameters (chronic liver disease, chemotherapy, etc.).

### Primary infection study (city laboratory database)

Patients were considered to have a primary infection if they had positive IgM anti-VCA without IgG anti-EBNA. It was impossible to exclude duplicate entries or to assess the kinetics of the serological results (S2 Fig).

### Ethical considerations

Approval was obtained on 2016-04-19 from the local ethical committee (CECIC Rhône-Alpes-Auvergne, Clermont-Ferrand, IRB 5891). Given the retrospective use of these results, we did not seek the informed consent of sampled patients, after discussion with this ethical committee.

### Statistical analysis

We used the Mann–Whitney to compare two sets of quantitative variables, the Spearman correlation test to explore correlations between continuous variables, and the Chi2 test for categorical variables, with a significance level of 0.05. Multivariable logistic regression was used to determine whether the period was associated with EBV seroprevalence after adjustment for age group and sex.

## Results

The main characteristics of our 4 populations (2 seroprevalence study populations composed of the hospital and city laboratory databases; 2 PI study populations composed of the hospital and city laboratory databases) are shown in Table 2. Concerning the seroprevalence study, hospital patients were significantly older than city laboratory patients (42.0±23.8 vs. 30.0±18.5, p<0.01). Concerning the PI study, hospital patients were significantly younger than city laboratory patients (14.6±11.5 vs. 18±10.6, p<0.01).

**Table 2:** Characteristics of the studied populations.

	Seroprevalence study			Primary infection study		
	Hospital database	City laboratory database	p	Hospital database	City laboratory database	p
**number**	42880	25094	/	636	2085	/
**>19 year old, n (%)**	33654 (78.5)	16444 (65.5)	<0.01	186 (29.2)	738 (35.4)	<0.01
**Mean age ±SD**	42.0±23.8	30.0±18.5	<0.01	14.6±11.5	18.0±10.6	<0.01
**Year of sample**	2001–2015	2008–2015	/	2000–2016	2008–2015	/
**% seropositivity**	88.3	82.0	<0.01	/		
**% of primary infection**	/			1.4	7.7	<0.01

SD, Standard deviation.

### Seroprevalence study

**Fig 1:**
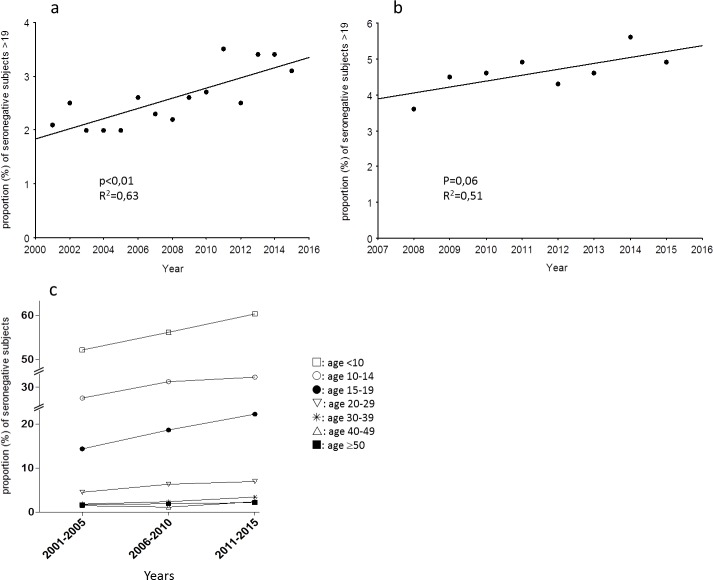
**a, b. Seroprevalence of EBV infection.** Proportion of seronegative patients older than 19 during the studied period in the hospital database (a) and the city laboratory database (b). (c) proportion of seronegative patients in 3 periods (2001–2005, 2005–2010, 2011–2015) for 6 age classes (<10, 10–14, 15–19, 20–29, 30–39, 40–49, and ≥50 years) in the hospital database.

We analyzed the seroprevalence in the population of patients aged 20 and over. In the hospital population, we observed a significant increase in the proportion of EBV seronegative samples for the years 2001–2015 (Fig 1A). In this population, 2.1%, 2.5%, and 3.1% of the patients were seronegative for EBV in the years 2001–2005, 2006–2010, and 2011–2015, respectively (p<0.01). When considering 6 age classes (Fig 1C), this increase in the proportion of seronegative patients was significant for age classes <10 years (p<0.01), 15–19 years (p<0.01), 20–29 years (p<0.01), and 30–39 years (p<0.01).

Moreover, in the hospital database, after adjustment for age group and sex, periods 2010–2011 (OR = 0.7 [95%CI: 0.5–0.9]) and 2014–2015 (OR = 0.7 [95%CI: 0.5–0.9]) were significantly associated with a decrease of EBV seroprevalence when compared with period 2001–2005.

The same trend was observed (p = 0.06) in the city laboratory population (Fig 1B). In this population, 4.3% and 4.9% of the patients aged 20 and over were seronegative for EBV in the years 2008–2010 and 2011–2015, respectively.

### Primary infection study: Age at PI

**Fig 2:**
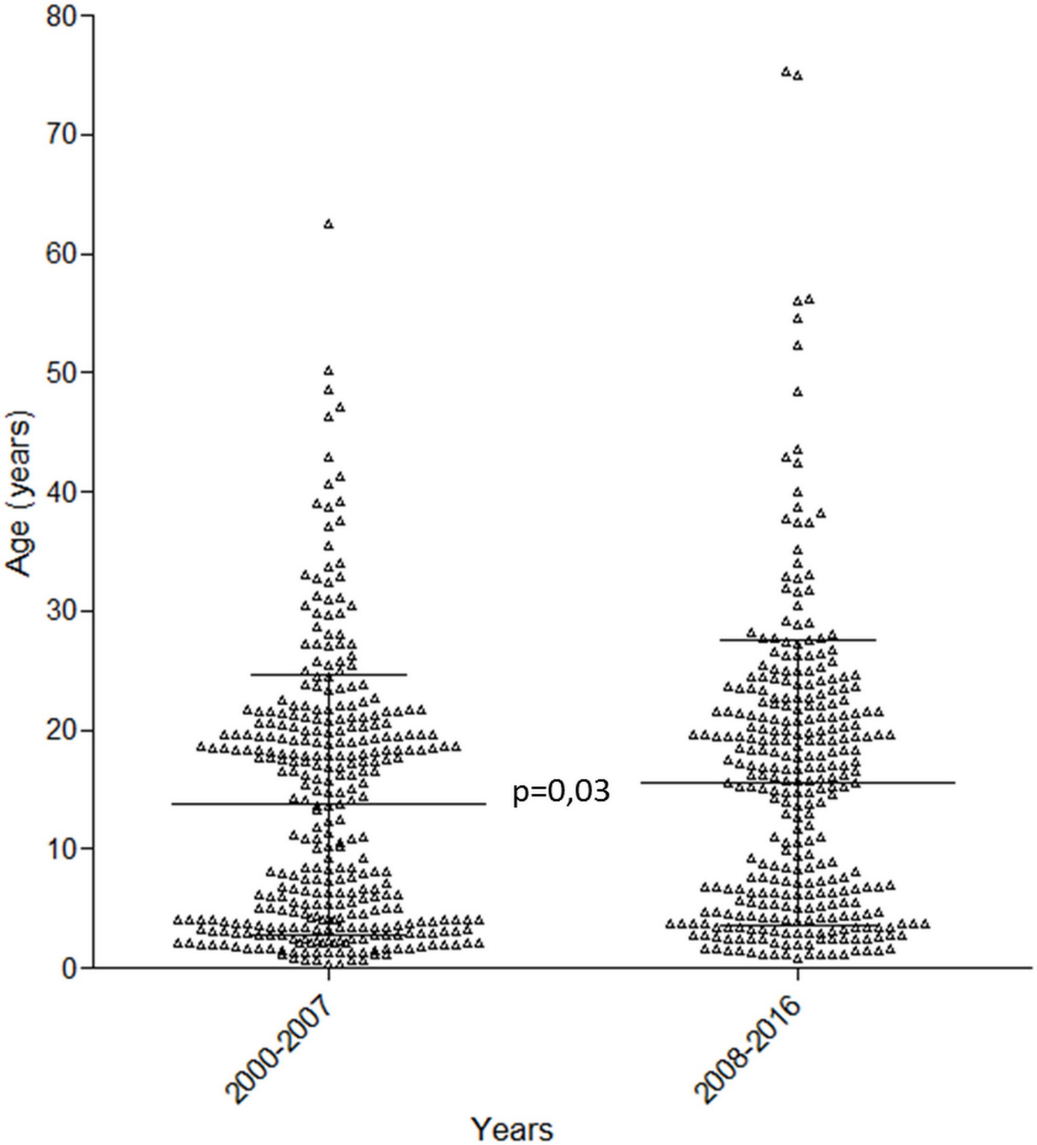
Age at EBV PI. Age of patients with EBV PI in the hospital database for the years 2000–2007 and 2008–2016 (lines: mean±SD).

In the hospital population, the age at PI was significantly higher in the years 2008–2016 than in the years 2000–2007 (15.6±12.0 vs. 13.7±11.0, p = 0.03) (Fig 2). We made the same observation when we considered only the 313 patients who tested positive for heterophile antibodies (18.2±10.4 vs. 13.9±11.1, p<0.01). We also observed a trend toward a positive weak correlation between age and the year of EBV PI diagnosis (p = 0.05, R^2^ = 0.06). No increase of the number of PI diagnoses was observed over the years. Noteworthy, no evolution of the age was seen in the larger population of the seroprevalence study (mean age for the years 2001–2007 and 2008–2015: 41.8±23, 42.0±24, respectively; p = 0.16), suggesting that the increasing of the age at PI was not due to an increasing of age of the global population.

For the city laboratory population, the age of patients at PI before and after 2012 were not significantly different (18.0±10.1 vs. 17.9±10.9, p = 0.28).

### Primary infection study: Age and intensity of PI (hospital database)

**Fig 3:**
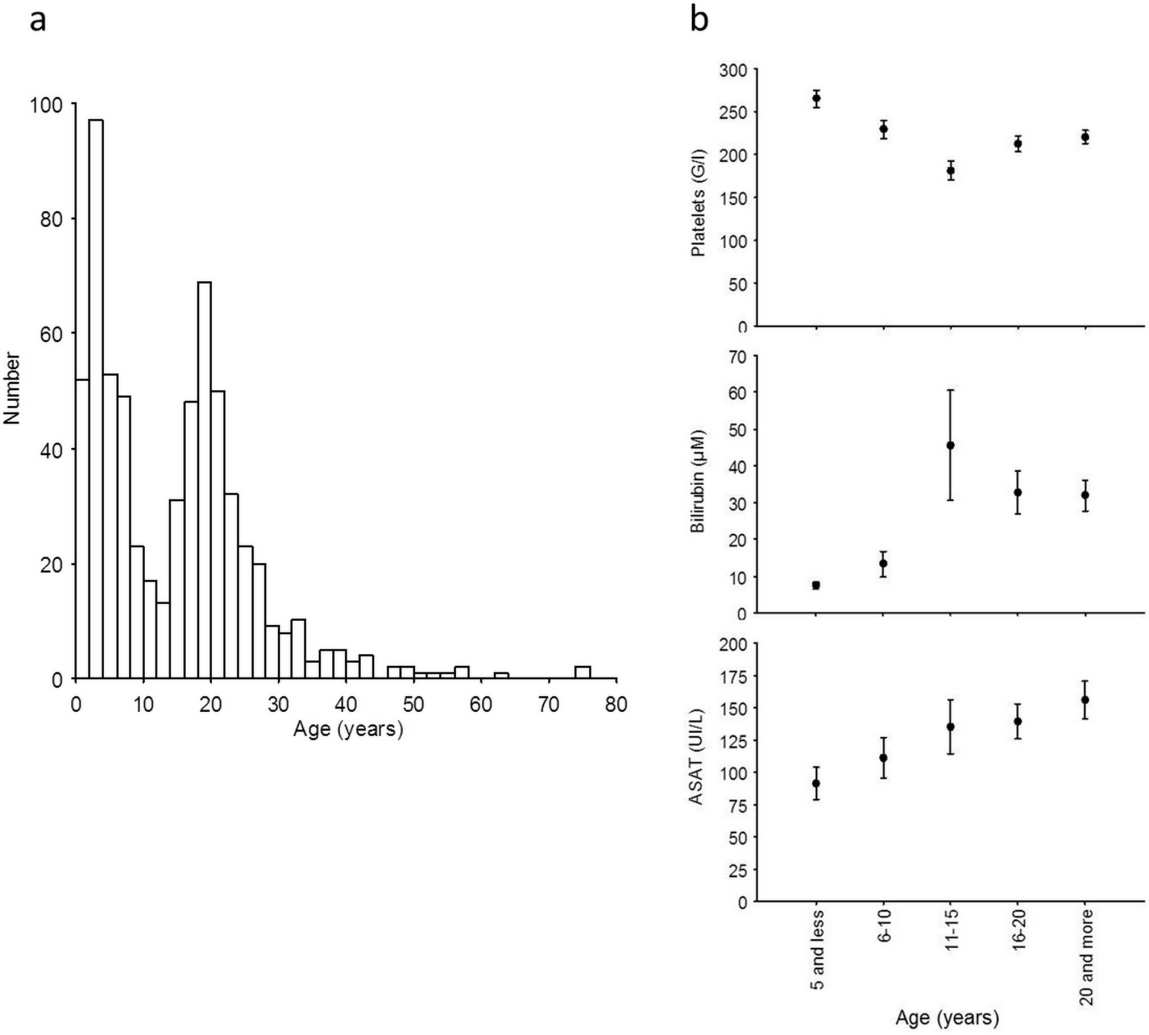
a) Age distibution of patients with EBV PI in the hospital database. b) Mean (± standard error) of blood platelet, ASAT, and bilirubin levels for 5 age groups during primary EBV infection.

Fig 3A shows the bimodal age distribution of the population of patients with PI, with a peak before and after 13 years.

Because of this bimodal distribution, we compared the characteristics of patients younger than and older than 13. The hospital stay length was not different in these 2 populations. Conversely, several biological parameters differed significantly between these 2 populations (Table 3), the older exhibiting higher ASAT, ALAT, γGT, and bilirubin blood level and lower thrombocyte level.

**Table 3:** Differences in various parameters for patients younger or older than 13.

Data	Age	Mean	Median	SD	p-value
**ASAT (UI/L)**	< 13 years old	97.8	56	102.2	<0.01
	≥ 13 years old	148.4	104	142.1	
**ALAT (UI/L)**	< 13 years old	112.1	50	135.8	<0.01
	≥ 13 years old	228.9	164	220.3	
**γGT (UI/L)**	< 13 years old	78.6	37	87.4	<0.01
	≥ 13 years old	221.2	142	232.1	
**Bilirubin (μmol/L**	< 13 years old	9.3	6	9.1	<0.01
	≥ 13 years old	33.9	13	45.3	
**C-reactive protein (mg/L)**	< 13 years old	33.5	17	45.9	0.10
	≥ 13 years old	37	25	45.9	
**Neutrophils (G/L)**	< 13 years old	4.1	3.5	2.8	0.71
	≥ 13 years old	3.9	3.2	2.4	
**Lymphocytes (G/L)**	< 13 years old	6.4	5.4	4.1	<0.01
	≥ 13 years old	4.8	4	3.2	
**Lymphocytes (%)**	< 13 years old	50.9	51	17.1	<0.01
	≥ 13 years old	45.3	45.8	15.1	
**Activated (atypical) lymphocytes (%)**	< 13 years old	6.1	4	5.6	0.24
	≥ 13 years old	6.8	5	6	
**Platelets (G/L)**	< 13 years old	251.8	115	238	<0.01
	≥ 13 years old	212.6	90.6	210	
**Hospital stay lenght (d)**	< 13 years old	2.2	1	2.9	0.10
	≥ 13 years old	3	1	3.4	

ALAT, alanine aminotransferase; ASAT, aspartate aminotransferase; γGT, gamma-glutamyl-transferase

Moreover, we observed a positive significant correlation between age (as a continuous variable) and ASAT, ALAT, γGT, and bilirubin levels (p<0.01), and a significant negative correlation between age and platelet counts (p<0.01). Consistently, a platelet count under 150 G/l was found in 15.5% and 23.2% of patients younger and older than 13, respectively (p = 0.04); an ASAT level of above 150 UI/l was found in 18.8% and 37.5% of patients younger and older than 13, respectively (p<0.01). Fig 3B shows the mean platelet, ASAT, and bilirubin levels for 5 age groups (means +/- SEM). We did not observe any correlation between age and the duration of hospitalization.

## Discussion

In the last decades, several works have reported changes in the epidemiology of EBV infection. For example, in Tokyo, Japan, and its neighboring prefectures, seroprevalence in 5-to-7-year-old children was higher than 80% before the early 1990s, whereas it decreased to 59% in the years 1995–1999 [5]. In the USA, seroprevalence in 6-to-19-year-olds declined from 72% in 2003–2004 to 65% in 2009–2010 [4]. Moreover, a study conducted in England and Wales suggests that the age at EBV PI is increasing [7]. We therefore aimed to determine whether the epidemiology of EBV infection has evolved over the last years in the Isère department, France.

To this end, we explored 2 large serological test databases. We found that the age at PI has risen over the last 15 years for the studied population of patients at Grenoble University Hospital: consistently, EBV seroprevalence decreased significantly in patients aged 20 and over. The fact that the same trend (although nonsignificant) for seroprevalence was also observed in the city laboratory database suggests that this result cannot be attributed to a selection bias. Moreover, this seroprevalence evolution was confirmed in a multivariable logistic regression analysis after adjustment for age group; this indicates that this observation is not due to a modification in the age of the general sampled population. Several factors may be responsible for this age shift. First, rising hygiene levels in developed countries might contribute to decreasing the transmission of the virus during early childhood. This hypothesis has also been proposed to explain the decrease in other herpes viruses such as cytomegalovirus [8] and *Herpes simplex* virus 1 [9]. Second, family size likely has an effect on age at EBV PI, as increasing sibship size reduces the risk of IM in adolescence [10]. Large families are becoming rarer in France, which could contribute to the decrease in EBV seroprevalence. Finally, changes in other factors such as the practice of breastfeeding infants [11], the organization of child day care, and an increased use of alcohol-based hand sanitizers in schools, may also lower the early transmission of EBV.

We also aimed to determine whether age of onset and intensity of EBV PI manifestations were correlated. We observed this correlation with surrogates of cholestasis and liver cytolysis, and with thrombopenia. Some studies had already reported that EBV-induced hepatitis is more intense in people aged 15 and over than in children under the age of 7 [12] and that EBV PI-associated cytolysis occurs less frequently in children than in adolescents [13]. Several hypotheses have been proposed to explain the link between age and EBV PI intensity. First, infection of B lymphocytes by EBV reproduces the activation process induced by the first contact between a naïve B cell and the antigen it is specific to, leading to the secretion of high amounts of immunoglobulins with extremely varied specificities. Moreover, the cellular immune response to EBV-infected B lymphocytes is particularly intense and may drive an overwhelming activation of T lymphocytes specific to non-EBV targets [14]. Such an intense and broad activation of both humoral and cellular immune responses may lead to inflammatory lesions in various organs, particularly in people who previously developed a vast immune repertoire, i.e. in adolescents and adults rather than in infants [15]. Second, EBV shedding in saliva is a persistent (although intermittent) and sometimes intense phenomenon in the months following EBV PI [16,17]. Because adolescents and adults are presumed to engage in deep kissing (a risk factor for EBV PI [18]) more frequently than children, the former may be exposed to massive amounts of virions, and therefore develop a more intense form of the disease.

Taken together, our data suggest that the incidence of IM is likely to rise, and that primary care physicians will be faced with more intense forms of IM in the coming years. This is consistent with two French studies [19,20] that described an increase in the number of IM patients requiring ICU hospitalization. Age shift may become apparent not only during the acute phase of IM, but also in the following weeks/months, given that IM has a substantial impact on academic performance, physical exercise, and social activities [21]. Moreover, IM itself is not the only concern. Indeed, IM is a risk factor for Hodgkin's disease (HD) [22] and multiple sclerosis (MS) [23]. The fact that EBV is an oncovirus for B lymphocytes partially explains the incidence peak of HD observed 2 to 3 years after IM. The link between MS and EBV is epidemiologically established (virtually all patients with MS are EBV infected, and a meta-analysis has shown that IM itself is a risk factor for MS [24]). However, the mechanisms involved remain obscure. A higher incidence of IM could therefore lead to more frequent occurrences of secondary MS and HD.

Several research groups have attempted to create an EBV vaccine, in particular to prevent the long-term consequences of EBV infection. Most of these vaccines used viral gp350 as a target; they have thus far failed to prevent EBV infection, although some data suggest that PI was more frequently asymptomatic in vaccinated people [25]. There may be growing interest in such a vaccine if the age at PI increases, and the vaccine could then be proposed, for example, to seronegative 13-year-old children.

Our study had several limitations. The major one results from the studied population. Indeed, we only included patients who had undergone EBV serological testing; therefore, our 2 populations (hospital population, city laboratory population) were not strictly representative of the global population. Different prevalence results could be observed if every healthy person in the population had received yearly EBV serological screening. As such a global screening is not performed, we chose to exploit these two data sets; however, the serologic tests were perform in a population with a suspected illness, and the trends we observed may differ in a healthy population. Second, the city laboratory database only contained 8 years of records (2008–2015). This relatively short period may explain the nonsignificant results. Third, the diagnosis of PI may not always be accurate. Indeed, patients whose serological test results showed isolated IgM anti-VCA may not have an EBV PI. However, when we analyzed only the 49.2% of patients with heterophile antibodies (a more specific test), the age at EBV PI still differed significantly between the two periods (2000–2007 and 2008–2016). This shows that our results are not due to a lack of specificity. Moreover, we verified the serological kinetics and the medical history in the hospital database for all patients aged 50 and over at primary infection, leading to the exclusion of 44 patients. However, this exclusion process was not possible for the city laboratory database. Furthermore, we were unable to exclude duplicates for this population.

## Conclusion

The analysis of our data suggests that EBV infection in France is acquired at a later age than previously observed, and that the severity of the infection is correlated with the age at PI. Therefore, in the coming years, severe EBV PI is likely to become more frequent, as may be the case for late complications of IM. An EBV vaccine may become even more relevant if this trend can be confirmed.

## Supporting information

S1 Fig flow-chart of the selection of data for hospital database (PI: primary infection). (TIF)

S2 Fig flow-chart of the selection of data for city laboratory database (PI: primary infection). (TIF)
