# Supplementary figures and images for: Evolution of EBV seroprevalence and primary infection age in a French hospital and a city laboratory network, 2000–2016
Source: PLoS One. 2017 Apr 17;12(4):e0175574. doi: 10.1371/journal.pone.0175574 (PMC5393566; doi:10.1371/journal.pone.0175574)

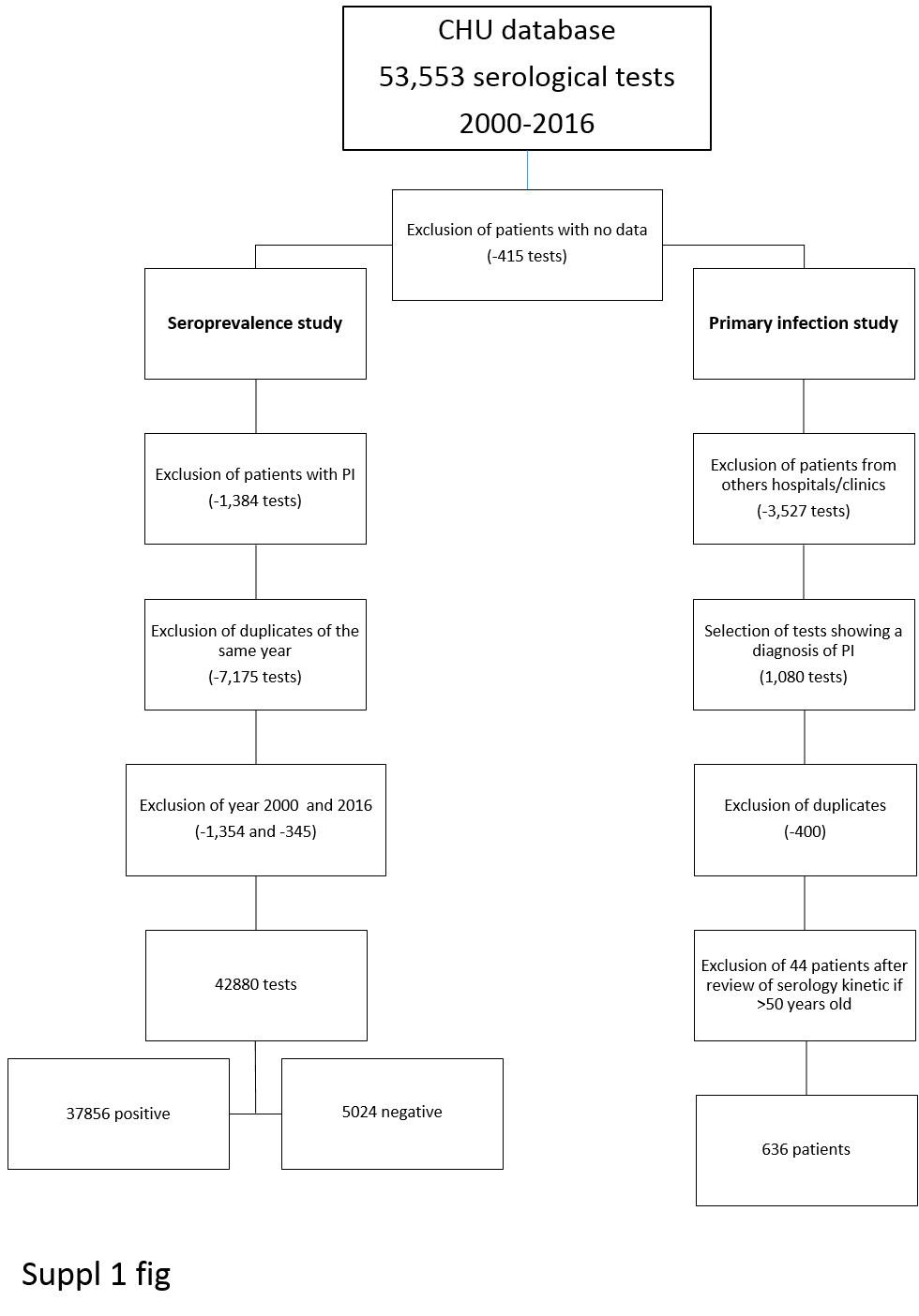

Supplement: S1 Fig — (TIF) [file pone.0175574.s001.tif]

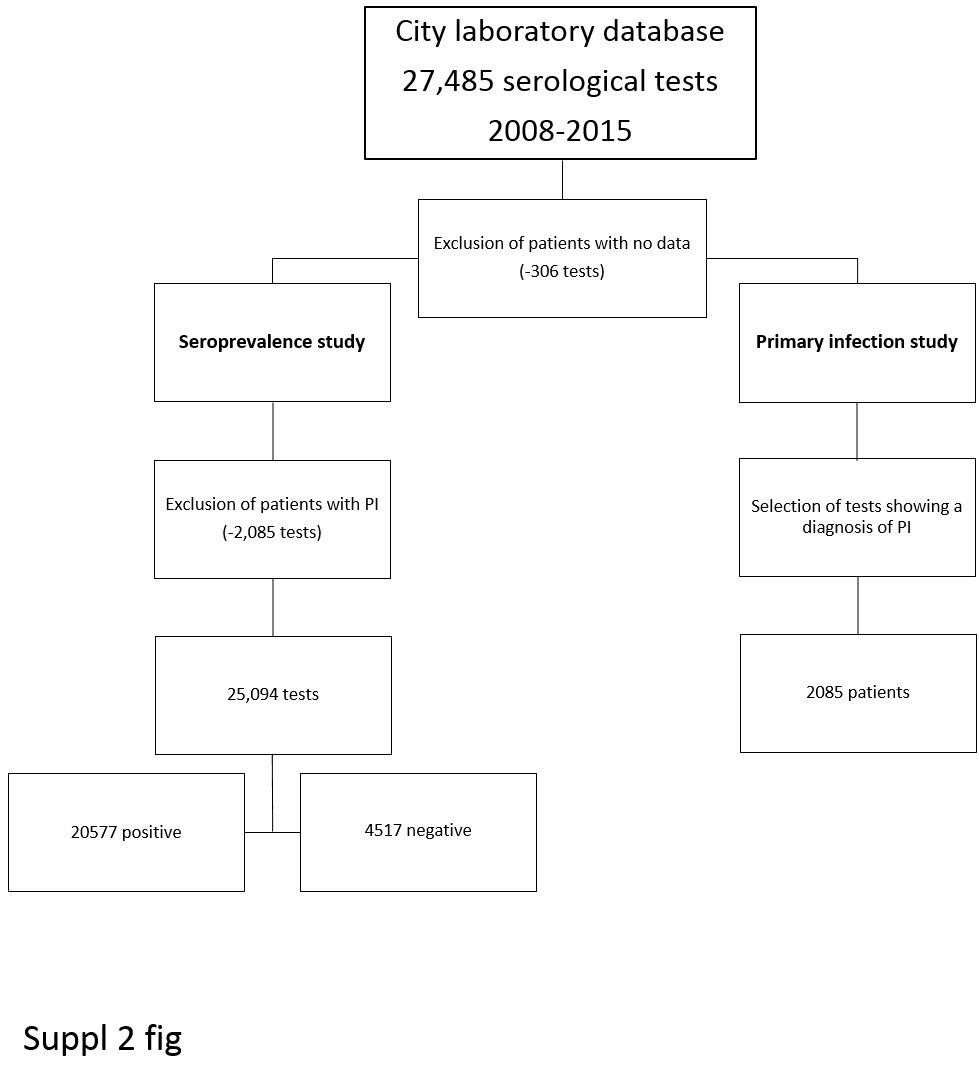

Supplement: S2 Fig — (TIF) [file pone.0175574.s002.tif]
